# Similarities between Ashi acupoints and myofascial trigger points: Exploring the relationship between body surface treatment points

**DOI:** 10.3389/fnins.2022.947884

**Published:** 2022-11-02

**Authors:** Seoyoung Lee, In-Seon Lee, Younbyoung Chae

**Affiliations:** ^1^Department of Science in Korean Medicine, Graduate School, Kyung Hee University, Seoul, South Korea; ^2^Acupuncture and Meridian Science Research Center, Kyung Hee University, Seoul, South Korea

**Keywords:** acupuncture, acupuncture points, Ashi point, myofascial trigger point, myofascial pain

## Abstract

Although acupuncture points and myofascial trigger points (TPs) are based in different medical fields, the two points share important attributes. We explored the relationship between acupuncture points and TPs based on their characteristics and the results of previous studies. We outlined the relationship between acupuncture points and TPs by examining their similarities and differences. Among the acupuncture point subgroups, TPs mostly corresponded to Ashi points. Based on the common features of TPs and Ashi points, we suggest that TPs are more closely related to Ashi points than to other acupoints. However, TPs also share some features, such as pain indication and location, with classical acupuncture points (CA) and extra acupuncture points (EA), which makes it difficult to elucidate their relationship with other subgroups. Therefore, we suggest to understand the relationship of CAs, EAs, Ashi points, and TPs. In this report, we concluded that concerning muscular pain symptoms Ashi points and TPs are indistinguishable.

## Introduction

The main assumption of biomedicine is that all human is biologically equivalent, and the development of biomedicine aided millions of lives for the past few centuries (Guta et al., 2017; Lock and Nguyen, 2018). However, limitations of modern biomedicine have led to a rapid interest in self-care and holistic medicine paradigm (Hastings, 2019). Whole person health emphasizes the wellbeing of each person and the mind-body connection, in addition, practices such as acupuncture, yoga, massage may give holistic benefits and improve wellbeing to individuals (Gould and MacPherson, 2001; Cavaye, 2012; Rosenbaum and Velde, 2016; Langevin, 2021b; Zheng and Zhou, 2022). Although acupuncture and massage treatment are known to improve wellbeing, the treatment area (e.g., acupuncture points) is also crucial.

**FIGURE 1:**
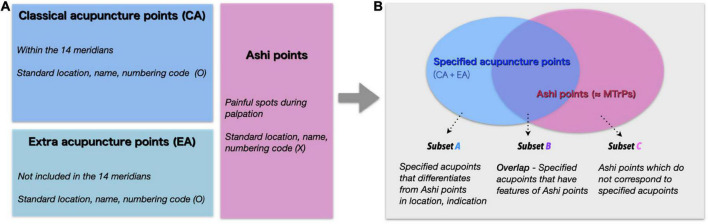
**(A)** The classical model of the relationship between acupuncture point subgroups. Classical acupuncture points (CA) in blue, extra acupuncture points (EA) in light blue, and Ashi points in pink. The blue and light blue areas are referred to as specified acupuncture points, comprising CA and EA. The three subgroups are considered mutually exclusive. **(B)** A new model of the relationship between specified acupuncture points and Ashi points. Subset A, shaded only in light blue, refers to the acupoints that are CA and EA, but not Ashi points. Subset B, which is filled in light purple (specified acupuncture points ∩ Ashi points), represents acupoints that can be CA or EA and Ashi points at the same time, for example when TPs are located in specified acupuncture points. Subset C, colored in pink, represents Ashi areas that are not defined as specified acupuncture points.

The traditional theory classifies acupuncture points into three categories: classical acupoints (CAs), extra acupoints (EAs), and Ashi points. There are 361 CAs along the 14 meridians, mainly indicated for visceral and meridian system disorders. CAs are the most important acupuncture points with 2,500 years of history (Dorsher, 2008b). EAs are acupoints that are not part of the 14 meridians, but are distinguished by their unique therapeutic properties including unique indications and effective treatment actions (Hong, 2018). Points that are clinically important are newly added acupuncture points to EAs, and these EAs are usually off-channel (Dorsher, 2008b). For example, the EX-HN5 acupoint does not belong to the meridian system, but it is widely used to treat headaches and migraines (Lu et al., 2021). Both CAs and EAs, here after “specified acupuncture points,” have defined location based on anatomical landmarks (WHO Regional Office for the Western Pacific, 2008; Lin and Tung, 2013). Ashi points, on the other hand, do not have defined positions and are the points where the patient feels pain during palpatory examination. The term “Ashi” is thought to be a combination of “A,” representing the patient’s scream, and “shi” for the confirmation of the painful spot or palpation point (Zhao, 2010). As Ashi points have no defined anatomical location, all areas of the body surface can be possible points. Despite the unsettled location of Ashi points, they can have powerful therapeutic effects because they can reflect and treat various pathological states such as disorders of soft tissue and internal organ (Chen et al., 2017). The relationships between the acupuncture point groups are shown in Figure 1. Myofascial trigger points (TPs) are hyperirritable sites in the taut bands of skeletal muscles (Lavelle et al., 2007; Barbero et al., 2019). Tenderness at these nodules can cause muscle pain, weakness, spasms, and autonomic symptoms such as piloerection, vasoconstriction, hyperhidrosis, temperature changes, and a variety of somatovisceral responses (Mitidieri et al., 2020).

While the demand for personalized medicine is increasing in modern medicine, Ashi points and TPs may be helpful to treat patients in the perspective of personalized medicine (Zhang et al., 2012). The connective tissue hyperlaxity may be altered in myofascial pain patients by injuries or posture, therefore customized treatment may be effective (Langevin, 2021a). Although Ashi points and personalized prescription are commonly used in clinical settings, clinical trials are more likely to select fixed acupuncture treatment protocols which are comparatively easier in quality control (Fei et al., 2022). Instead of using standardized acupuncture points which are located mainly by anatomical structures, selecting Ashi and TPs focuses on the area that is most triggering to the patient. By these reasons, Ashi and TPs are closely related to personalized treatment compared to CAs and EAs. The patient’s perceived discomfort and complaint are more important in Ashi and TPs, thus the patient’s involvement in the treatment is more significant compared to fixed acupuncture prescriptions. This enhanced involvement can be connected to shared-decision making of treatment. Shared-decision making can influence treatment compliance, doctor-patient relationship and treatment satisfaction (Siminoff and Step, 2005; Milky and Thomas, 2020; Deniz et al., 2021). These aspects of prescribing Ashi points may influence treatment effect, satisfaction, doctor-patient relation, etc., however, was not reported or studied yet.

Acupuncture points and TPs have striking similarities, despite their origins in two different medical fields: traditional East Asian medicine and Western medicine. Melzack et al. (1977) identified a strong link between acupuncture points and myofascial TPs (71% correspondence) in terms of spatial distribution and pain patterns. Dorsher (2008a) proposed the anatomic, clinical, and physiologic correspondence of common TPs and CAs, especially in pain disorders. However, Birch negated the claim of correspondence between TPs and CAs (Birch, 2003). The discussion of the relationship between TPs and acupuncture points raised significant viewpoints of both TPs and acupuncture points. However, no consent was approved after this debate and only a few studies have investigated this issue. In addition, Ashi points and TPs are both frequently used in clinical settings. A previous survey of 276 Korean medicine doctors on the treatment of sports injury patients showed that Ashi points were most commonly used (20.5%), following TPs (20.2%) (Ha et al., 2018). This study reflects the frequent usage of both Ashi points and TPs in clinical environment. Whereas Ashi points are commonly selected by physicians, consensus and researches of Ashi points are rare compared to TPs (Fernandez-de-Las-Penas and Dommerholt, 2018). Moreover, when the two treatments are presented together, it may be confusing to discriminate the two concepts. Thus, investigating the relationship of Ashi points and TPs is crucial.

Here, we examined the characteristics of Ashi points and TPs, and the similarities between them. We aimed to evaluate the relationship between these two concepts by evaluating their similarities and differences. Additionally, we suggest a new model in understanding the relationship of the body surface treatment points (CA, EA, Ashi points, and TPs).

### Similarities between Ashi points and myofascial trigger points

After more than two-decades since Melzack first suggested that acupuncture points and TPs represent the same phenomenon in different labels, Birch rejected the idea by stating that the acupuncture points used in the study did not have key features of TPs (Melzack et al., 1977; Birch, 2003). The features are as follows, acupuncture points should have pressure pain and should be used to treat muscular or pain symptoms by local point indication. Still he agreed that there may have correspondence between Ashi points and TPs (Birch, 2003). In fact, Ashi points are more similar to TPs than to other acupoints (Liu et al., 2016).

First, Ashi points and TPs are both tender points, and compression of both these points elicits a painful response. Furthermore, palpation is critical for the identification of these points, including the physician’s tactile sensation and the patient’s subjective sensations. The number of Ashi points and their locations can vary from person to person. Most importantly, Ashi and TP sites are targets for treatment and the mechanical stimulation of these points by acupuncture or dry needling elicits similar responses: a local twitch in TP treatment and *deqi* in Ashi point treatment (Chou et al., 2012).

However, there are some differences between the two systems. Since TPs are found on the taut bands of skeletal muscles, their location is more specific than Ashi points which can be located anywhere in the body surface (Alvarez and Rockwell, 2002; Brazkiewicz, 2020). Ashi points may be present in any superficial part of the body, regardless of muscle distribution. TPs may be determined as Ashi points based on a patient’s response, but Ashi points can reflect diseases from non-muscle regions (Chen et al., 2017). TPs are frequently found in pain disorders, such as myofascial pain syndrome, while Ashi points may be present in non-pain and non-muscular conditions, such as functional gastrointestinal disorders (Hwang et al., 2019; Jun et al., 2021).

Based on these similarities and differences, we suggest that TPs are muscle-specific Ashi points. We suggest to understand these two concepts together to prevent confusion among practitioners and researchers from different fields. Detection and treatment of Ashi points lack research, and previous knowledge from TPs may help practice and research of Ashi points.

### Relationship between myofascial trigger points and acupuncture point subgroups

It has been debated which acupuncture subgroups are most related to TPs (Birch, 2003, 2008; Dorsher, 2008a). Many Ashi point locations coincide other acupuncture sites (CAs and EAs), possibly because physicians commonly test pressure pain/discomfort at CAs and EAs. Indeed, pressure-induced pain and discomfort is found in the great majority of CAs and EAs, not just in Ashi points. These characteristics make it difficult to distinguish between acupuncture subgroups and TPs. Previous viewpoints of the relationship between the three acupuncture categories classify each category mutually exclusive. However, we newly present a model of the relationship between specified acupuncture points and Ashi points, where specified acupuncture points and Ashi points share characteristics. Both models of the relationship of the subgroup of acupuncture points are shown in Figure 1.

Subset A (CA + EA) refers to non-Ashi acupoints which have fixed anatomical location. Histological investigations have shown that neurovascular bundles, comprising A and C fibers, are concentrated in these places, and these acupoints may be excitable muscle/skin nerve complexes with a high density of nerve endings (Li et al., 2004). *Deqi* sensations, such as soreness and dull pain, involve slow-conducting Aδ and C fibers, and are important for the therapeutic effects of acupuncture (Zhang et al., 2013). Furthermore, subset A includes acupoints that are not solely indicated for local pain. These include major acupoints used not only for the local treatment of acupuncture point pain, but also for remote effects *via* the meridian system or systemic neurological effects (Lee and Chae, 2021; Lee et al., 2022). For example, PC6 located in the forearm area can be frequently used to treat functional gastrointestinal diseases. Recent RCT on patients with irritable bowel syndrome with constipation showed that transcutaneous electrical acustimulation on PC6 and ST36 resulted to improvement in constipation and abdominal pain (Huang et al., 2022).

Subset B refers to areas where specified acupuncture points (CA + EA) and Ashi points coincide. In previous studies comparing CAs and TPs, a high proportion of TPs coincided with CAs. Subset B includes sites where TPs are found in specified acupuncture points. TPs were shown to be similar to specific acupuncture points based on myofascial referred pain data from the Trigger Point Manual (Dorsher, 2009). In addition, pressure pain may be considered when finding CAs or EAs. Notably, pressure pain and sensitivity can be indicators of specified acupuncture locations in addition to anatomical locations. However, tenderness is not always important for acupuncture. Tenderness of acupuncture points is useful only for locating subset B acupoints, such as SP10, LI10, and EX-HN5, which are located in the vastus medialis, brachioradialis, and temporalis muscles (Vicente-Barrero et al., 2012; Chang et al., 2020; Polat et al., 2021). The locations of these acupoints commonly overlap with TPs of these muscles, showing similarities between TPs and specified acupoint locations. While these acupuncture points also have distal effects based on the traditional meridian, such as the LI10 to treat diseases in the large intestine and other digestive systems. Recent study showed that in mice with peptic ulcer disease, electroacupuncture in LI10 resulted to a significant reduce in the injury of the gastroduodenal mucosa and an increase in the diversity of gastric microbiota (Li et al., 2022).

**FIGURE 2:**
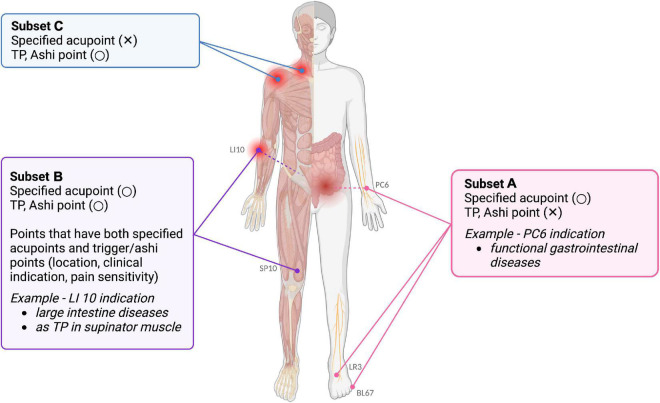
Examples of subsets A, B, and C on a human body template. Subset A consists of specified acupoints, but not TPs or Ashi points. Neurovascular bundles, comprising A and C fibers, are concentrated at these places. For instance, acupoint PC6 is known to treat functional gastrointestinal diseases. Subset B includes areas with common features of specified acupoints, TPs, and Ashi points. These common features include similar locations, clinical indications, and pain sensitivity. For example, acupoint LI10 in the large intestine meridian can treat diseases of the large intestine, while it can serve as TP of the supraspinatus muscle. Subset C comprises points that are not defined as specified acupoints, but are TPs or Ashi points. Muscles and fasciae are the main targets of subset B and C treatment. The image was created using BioRender (www.biorender.com).

Subset C includes Ashi points, more specifically TPs (muscular Ashi points) and non-muscular Ashi points. A majority of TPs and specified acupoints have shared locations; however, some areas, such as the TP of the anterior deltoid muscle, do not have a corresponding specified acupuncture point (Shah et al., 2015). Since TPs and Ashi points can occur in different places, there are specific areas where TPs and Ashi points are incongruent to specified acupoints. TPs are hypothesized to be initiated when a sensitive locus (a nociceptor) and an active locus (motor endplate) co-occur, and this sensitive locus is thought to be concentrated at the skeletal muscle TP (Lavelle et al., 2007; Fernandez-de-Las-Penas and Dommerholt, 2018). Figure 2 further illustrates the subset on the human body template with examples.

The relationship between acupuncture points and TPs has long been a matter of debate (Melzack et al., 1977; Birch, 2003, 2008; Dorsher, 2008a). Ashi points derived from Traditional Chinese medicine (TCM) while, the history of TPs starts from muscle pain disorders from western medicine (Shah et al., 2015). Due to this disparity in their backgrounds, reconciliation of the two terms has proven problematic. In this paper, we examined the association between Ashi points and TPs by clarifying the relationship between the three subsets of acupuncture points. Acupuncture points were separated into three groups to investigate their relationship with TPs. Based on the similarities and differences, we suggest that TPs can be considered a specific form of Ashi points because of the similar underlying concepts. We expect researchers and practitioners to understand these two concepts together to reduce the confusion between different fields and to apply research findings and techniques of each concept together.

## Discussion

In this study, we elaborated the similarities and differences between CAs, EAs, and TPs. Acupuncture is extensively used worldwide, and more than 4,000 acupuncture trials have been reported over the past 5 years (Fei et al., 2022). However, most studies have investigated CAs and have overlooked Ashi points. A previous study that used data mining of randomized controlled clinical trials (RCT) for pain control demonstrated a high frequency SP6, ST36, LI4, and LR3 point selection (Hwang et al., 2020). The demand for personalized treatment is growing, and precision medicine has initiated the shift from disease-based medicine to personalized medicine (Yuan, 2021). Individualized treatment *via* pattern identification is one of the key features of TCM (Wang et al., 2018). Even though all acupuncture points can have an individualized approach, physicians have to choose from the fixed acupuncture points. The connective tissue hyperlaxity may be altered in myofascial pain patients by injuries or posture, therefore customized treatment may be effective (Langevin, 2021a). However, each patient may have different conditions–e.g., different connectivity alteration from different pathological history–and the treatment location may not exist in preexisting acupoints such as CAs or EAs. In addition, a recent review pointed out that clinical trials of acupuncture tend to choose fixed acupuncture treatment protocols instead of treatment based on symptom differentiation (Fei et al., 2022). Therefore, Ashi and TPs can be considered more personalized compared to CAs or EAs.

The personalized features of TPs and Ashi points can meet these demands in conditions involving pain, muscles, and fascia. Myofascial pain is one of the most common conditions of chronic pain (Fricton, 2016). A recent review on musculoskeletal pain suggested that mind-body approaches including acupuncture and massage may help these conditions by prevention through awaking awareness and revise faulty movement habits (Langevin, 2021c). In addition, the literature proposed the importance of personalized medicine in non-pharmacological treatments. Multi-disciplinary researches are important for better understanding of myofascial pain and to establish a quantitative method to measure the muscle and fascia (Langevin, 2021a). Further understanding of acupuncture subgroups and their relationship with TPs can reduce the confusion of two similar concepts, and provide new perspectives on both terms.

We further suggest studies on the sensory and emotional properties involved in treatment of Ashi points and TPs. Earlier studies have observed the sensory characteristics of TPs such as lower pressure pain threshold (Hong, 1998). Multidisciplinary approaches are attempting to objectively measure the properties of TPs and further studies investigating the sensory characteristics of TPs can provide hints to measure the properties of the muscle and fascia (Langevin, 2021a). Previous research has explored the treatment effect of TPs in terms of reducing pain, increased range of motion and blood flow (Moraska et al., 2013; Cagnie et al., 2015). However, studies have not yet investigated on the emotional and neural components involved in the treatment of Ashi point and TP. We presume that individuals may seek more treatment on their own Ashi and TPs. In addition, we expect enhanced patient-physician relationship during the detection and treatment of Ashi points and TPs, as physicians would focus comparatively more on the current discomfort during the treatment. Also, instead of the doctor solely choosing from the standardized acupuncture points, finding the point from palpation and patients’ complaint may be closer to shared-decision making process of treatment.

In summary, Ashi points and TPs share common features, and we suggest that Ashi points are a broader category which include TPs as a subtype (muscular Ashi points). Furthermore, we explored the relationship between specified acupuncture points and Ashi points. In order to understand this relationship in a simple matter, we classified the relationship between specified acupuncture points and Ashi points into three subsets. Notably, we suggest that some Ashi points can coincide with specified acupuncture points. Additional studies and in-depth expert discussions are required to clarify the relationship between these points to improve their clinical implications and strength of evidence.

## Data availability statement

The original contributions presented in this study are included in the article/supplementary material, further inquiries can be directed to the corresponding authors.

## Author contributions

SL wrote the first draft of the manuscript. All authors conceived and revised the manuscript and approved the final version.
